# A multicentre and multi-national evaluation of the accuracy of quantitative Lu-177 SPECT/CT imaging performed within the MRTDosimetry project

**DOI:** 10.1186/s40658-021-00397-0

**Published:** 2021-07-23

**Authors:** Johannes Tran-Gia, Ana M. Denis-Bacelar, Kelley M. Ferreira, Andrew P. Robinson, Nicholas Calvert, Andrew J. Fenwick, Domenico Finocchiaro, Federica Fioroni, Elisa Grassi, Warda Heetun, Stephanie J. Jewitt, Maria Kotzassarlidou, Michael Ljungberg, Daniel R. McGowan, Nathaniel Scott, James Scuffham, Katarina Sjögreen Gleisner, Jill Tipping, Jill Wevrett, The MRTDosimetry Collaboration, Manuel Bardiès, Salvatore Berenato, Ilias Bilas, Christophe Bobin, Marco Capogni, Maxime Chauvin, Sean Collins, Maurice Cox, Jérémie Dabin, Marco D’Arienzo, Johan Gustafsson, Aida Hallam, Theodoros Kalathas, Gunjan Kayal, Giuseppe Lorusso, Franz-Josef Maringer, Darren Morgan, Vere Smyth, Jaroslav Šolc, Ludmila Štemberková, Lara Struelens, Alex Vergara-Gil, Hannah Wiedner, Michael Lassmann

**Affiliations:** 1 Department of Nuclear Medicine, University of Würzburg, Oberdürrbacher Str. 6, 97080 Würzburg, Germany; 2 National Physical Laboratory, Teddington, UK; 3 Christie Medical Physics and Engineering (CMPE), The Christie NHS Foundation Trust, Manchester, UK; 4 The University of Manchester, Manchester, UK; 5 Cardiff University, Cardiff, UK; 6 Medical Physics Unit, Azienda Unità Sanitaria Locale di Reggio Emilia—IRCCS, Reggio Emilia, Italy; 7 Department of Physics and Astronomy, University of Bologna, Bologna, Italy; 8 Radiation Physics and Protection, Churchill Hospital, Oxford University Hospitals NHS Foundation Trust, Oxford, UK; 9 Nuclear Medicine Department, “THEAGENIO” Anticancer Hospital, Thessaloniki, Greece; 10 Medical Radiation Physics, Lund, Lund University, Lund, Sweden; 11 Department of Oncology, University of Oxford, Oxford, UK; 12 Royal Surrey County Hospital, Guildford, UK; 13 Department of Physics, University of Surrey, Guildford, UK

**Keywords:** Quantitative SPECT/CT, ^177^Lu SPECT/CT imaging, Standardization of SPECT/CT imaging, Harmonization of SPECT/CT imaging, International multicenter comparison exercise, Traceability of SPECT/CT imaging, Molecular radiotherapy (MRT), 3D printing, Phantom

## Abstract

**Purpose:**

Patient-specific dosimetry is required to ensure the safety of molecular radiotherapy and to predict response. Dosimetry involves several steps, the first of which is the determination of the activity of the radiopharmaceutical taken up by an organ/lesion over time. As uncertainties propagate along each of the subsequent steps (integration of the time–activity curve, absorbed dose calculation), establishing a reliable activity quantification is essential. The MRTDosimetry project was a European initiative to bring together expertise in metrology and nuclear medicine research, with one main goal of standardizing quantitative ^177^Lu SPECT/CT imaging based on a calibration protocol developed and tested in a multicentre inter-comparison. This study presents the setup and results of this comparison exercise.

**Methods:**

The inter-comparison included nine SPECT/CT systems. Each site performed a set of three measurements with the same setup (system, acquisition and reconstruction): (1) Determination of an image calibration for conversion from counts to activity concentration (large cylinder phantom), (2) determination of recovery coefficients for partial volume correction (IEC NEMA PET body phantom with sphere inserts), (3) validation of the established quantitative imaging setup using a 3D printed two-organ phantom (ICRP110-based kidney and spleen). In contrast to previous efforts, traceability of the activity measurement was required for each participant, and all participants were asked to calculate uncertainties for their SPECT-based activities.

**Results:**

Similar combinations of imaging system and reconstruction lead to similar image calibration factors. The activity ratio results of the anthropomorphic phantom validation demonstrate significant harmonization of quantitative imaging performance between the sites with all sites falling within one standard deviation of the mean values for all inserts. Activity recovery was underestimated for total kidney, spleen, and kidney cortex, while it was overestimated for the medulla.

**Conclusion:**

This international comparison exercise demonstrates that harmonization of quantitative SPECT/CT is feasible when following very specific instructions of a dedicated calibration protocol, as developed within the MRTDosimetry project. While quantitative imaging performance demonstrates significant harmonization, an over- and underestimation of the activity recovery highlights the limitations of any partial volume correction in the presence of spill-in and spill-out between two adjacent volumes of interests.

## Introduction

Tomographic molecular imaging methods such as single photon emission computed tomography in combination with x-ray computed tomography (SPECT/CT) provide powerful image quantification techniques with applications for diagnostics and therapy optimization [1–4]. Accurate quantitative imaging (QI) is an essential input to absorbed dose calculations in molecular radiotherapy (MRT), enabling an assessment of the activity distribution in a patient [5]. This, in turn, allows the calculation of absorbed doses to organs, tissues, or tumours of interest that can be exploited for optimizing the treatment.

In contrast to the more common qualitative use of SPECT/CT, quantitative imaging with SPECT/CT allows a direct measurement of the activity distribution within a given source region. Such direct measurement requires a calibration to relate the detected counts to the activity of a specific radionuclide, commonly defined by an image calibration factor (ICF) expressed in counts per second per MBq (cps/MBq). Calculation of an ICF typically involves the preparation of a test object (phantom) with a known activity concentration of a radionuclide for imaging on the SPECT/CT system to be calibrated [6]. This process can have multiple sources of uncertainty [7, 8] related to the accuracy and traceability of the phantom preparation, artifacts or errors in the SPECT/CT image reconstruction [9], and the choice of image reconstruction parameters.

The uncertainties in QI calibration, including uncertainties related to the determination of activity distributions in relatively small organs and tumour volumes [10], propagate directly to subsequent absorbed dose calculations [7, 11]. Accurate evaluation of the uncertainty on the complete measurement chain is therefore essential when optimizing therapies based on these calculations. For clinical trials involving dosimetry, the comparability of dosimetry results is of importance. Currently, the calibration of SPECT/CT QI is highly site-dependent, even among those using the same SPECT/CT systems and reconstruction parameters, and few efforts have been undertaken to establish traceability of activity quantification across sites. The importance of traceability for MRT dosimetry was highlighted in a recent review of multicentre studies on standardized quantitative imaging and dosimetry for radionuclide therapies by Lassmann et al. [12]. Only three studies describing the use of ^177^Lu were identified [13–15], of which only one made use of traceable activities [14].

The “Metrology for clinical implementation of dosimetry in molecular radiotherapy” project (MRTDosimetry) was a joint research project (JRP) within the European Metrology Programme for Innovation and Research (EMPIR), which ran for 3 years, finishing on 31 May 2019. This initiative brought together expertise in metrology and nuclear medicine research to address the problem of assessing the radiation absorbed dose to individual patients who are undergoing MRT. A main part of the project was the development of a protocol for commissioning and quality control of quantitative ^177^Lu SPECT/CT imaging, the feasibility of which was tested in a multicentre clinical SPECT/CT imaging comparison exercise among the partners of the consortium.

In this study, the results of the ^177^Lu SPECT/CT QI comparison exercise from the MRTDosimetry project are presented. The experimental protocol used to harmonize QI across the participating sites is outlined. The protocol includes (1) determination of an appropriate ICF, (2) correction of partial volume effects, and (3) validation of QI using a 3D printed two-organ phantom (based on kidney and spleen models from ICRP110 [16]). The results for activity recovery in realistic organ volumes are presented. The harmonization of image quantification between centres and the potential for the measurement protocol to be used for commissioning of quantitative ^177^Lu SPECT/CT imaging is discussed. To allow clinical centres to implement the techniques presented in this work and benchmark QI against these results, the standard operating procedure for the comparison exercise and the designs used for the phantom fabrication (in the STL file format) have been made available [17], and the designs used for the phantom fabrication (in the STL file format) have also been made available [17].

## Methods

A standard protocol was followed to calculate an image calibration factor, provide a correction for partial volume effects and to acquire SPECT/CT data of a custom-designed 3D printed two-organ phantom. A description of the comparison exercise and details of the data acquisition and image analysis are given in the following sections.

### Comparison exercise

Eight members of the MRTDosimetry consortium (Azienda Unità Sanitaria Locale di Reggio Emilia, The Christie NHS Foundation Trust, Lund University, National Physical Laboratory (NPL), Oxford University Hospitals NHS Foundation Trust, Royal Surrey County Hospital, “THEAGENIO” Anticancer Hospital, and University of Würzburg) participated in the comparison exercise. In total, nine systems were included in the study; details of the camera models and reconstruction software used for this study are given in Table 1.

**Table 1:** Details of SPECT/CT systems and reconstruction software used in the comparison exercise. *TEW*, triple-energy window; *ESSE,* effective scatter source estimation [18, 19]

Setup ID	Manufacturer	Model	CT slices	Crystal thickness	Reconstruction software	Scatter correction	Resolution recovery
S1	Siemens	Symbia T2	2	9.5 mm	Siemens e.soft	TEW	Yes
S2a	GE Healthcare	Discovery 670	16	9.5 mm	GE Xeleris	TEW	Yes
S2b	GE Healthcare	Discovery 670	16	9.5 mm	Hermes	TEW	Yes
S2c	GE Healthcare	Discovery 670	16	9.5 mm	Hermes	Monte Carlo	Yes
S3	GE Healthcare	Discovery 670	16	15.9 mm	In-House	ESSE	Yes
S4	Mediso	AnyScan SCP	16	9.5 mm	Nucline	TEW	No
S5	GE Healthcare	Discovery 670	16	9.5 mm	GE Xeleris	TEW	Yes
S6	GE Healthcare	Optima NM/CT 640	4	9.5 mm	GE Xeleris	TEW	Yes
S7	GE Healthcare	Optima NM/CT 640	4	9.5 mm	GE Xeleris	TEW	Yes
S8	Siemens	Symbia Intevo Bold	16	9.5 mm	Siemens e.soft	TEW	Yes
S9	Siemens	Symbia T2	2	15.9 mm	Siemens e.soft	TEW	Yes

When harmonizing results in any nuclear medicine comparison, it is essential to ensure that the various methods used at the sites for radionuclide activity measurement are all traceable to an appropriate primary standard for ^177^Lu. The methods used in this study included measurements with (i) a High Purity Germanium detector (HPGe) previously calibrated against primary standards, (ii) radionuclide calibrators and secondary standard ionization chambers previously calibrated against primary standards for the radionuclide of interest and a well-defined geometry [20, 21], and (iii) for sites where traceability had not previously been shown, a sample from a stock solutions was sent to National Physical Laboratory to proceed with calibration and ensure traceability to primary standards.

All participants submitted results for phantom activities and counts in volumes of interest for the three phantom measurements (described in the following sections) using a common reporting template for centralized analysis. For one site with access to a range of different reconstruction software (S2a–S2c), an individual dataset was submitted for each reconstruction setup. One site (S3) submitted an incomplete dataset, and these data were excluded from the comparison.

### Data acquisition

#### Image calibration factor for ^177^Lu

To determine the image calibration factor, a cylindrical phantom (Jaszczak phantom), with nominal volume 6.9 L [22], was filled with a uniform distribution of ^177^Lu (target activity of 400 MBq). To ensure a uniform activity distribution of ^177^Lu in the phantom, the use of a lutetium chloride carrier solution (10 μg·g^-1^ of inactive lutetium dissolved in 0.1 M hydrochloric acid) was recommended. The filling volume was determined by weighing (difference between filled and empty phantom). The dispensed activity by the participants varied between 387 and 410 MBq (average 400 MBq, standard deviation 11 MBq) at the start of the SPECT acquisitions. SPECT/CT data were acquired with the phantom positioned in the centre of the SPECT field of view using the acquisition parameters described in Table 2.

**Table 2:** SPECT/CT acquisition parameters

Collimator	Medium energy
Photopeak energy (keV)	208.4 ± 20.8 (20% width)^a^
Lower scatter energy (keV)	181.3 ± 6.3 (6% width)^b^
High scatter energy (keV)	235.5 ± 6.3 (6 % width)^b^
Flood uniformity	As per clinical imaging protocol for ^177^Lu
Energy calibration peak and source activity	208.4 keV (activity as per manufacturer recommendation)
Matrix size	128
SPECT movement	Body contour
Number of projections	120 (60 per detector)
Time/projection	60 s
Detector movement	Step-and-shoot
CT	Standard low-dose protocol

^a^According to [11], reasonable quantitative accuracy can be achieved using only the 208 keV peak or including the 113 keV peak for a medium energy collimator. ^b^ Scatter windows may be adjusted as required by a specific SPECT system or scatter correction method (as used for clinical measurements)

#### Partial volume correction

Resolution and partial volume effects were assessed using the six-sphere insert of the IEC NEMA PET body phantom (NEMA phantom) [23] with uniform activity distribution in the inserts and a water-filled background. Despite the relatively small volumes of these inserts, the NEMA phantom was chosen as it was readily available at all imaging centres. The inserts were filled with a uniform activity distribution of ^177^Lu (target activity concentration of 2.0 MBq/mL). The filling volume of each sphere was determined by weighing (difference between filled and empty phantom). The dispensed activity concentration by the participants for this phantom was between 1.8 MBq/mL and 2.3 MBq/mL (mean 2.1 MBq/mL, standard deviation 0.2 MBq/mL) at the start of the SPECT acquisition. SPECT/CT data were acquired with the phantom positioned in the centre of the SPECT field of view using the acquisition parameters described in Table 2.

#### 3D printed two-organ phantom

**Fig. 1:**
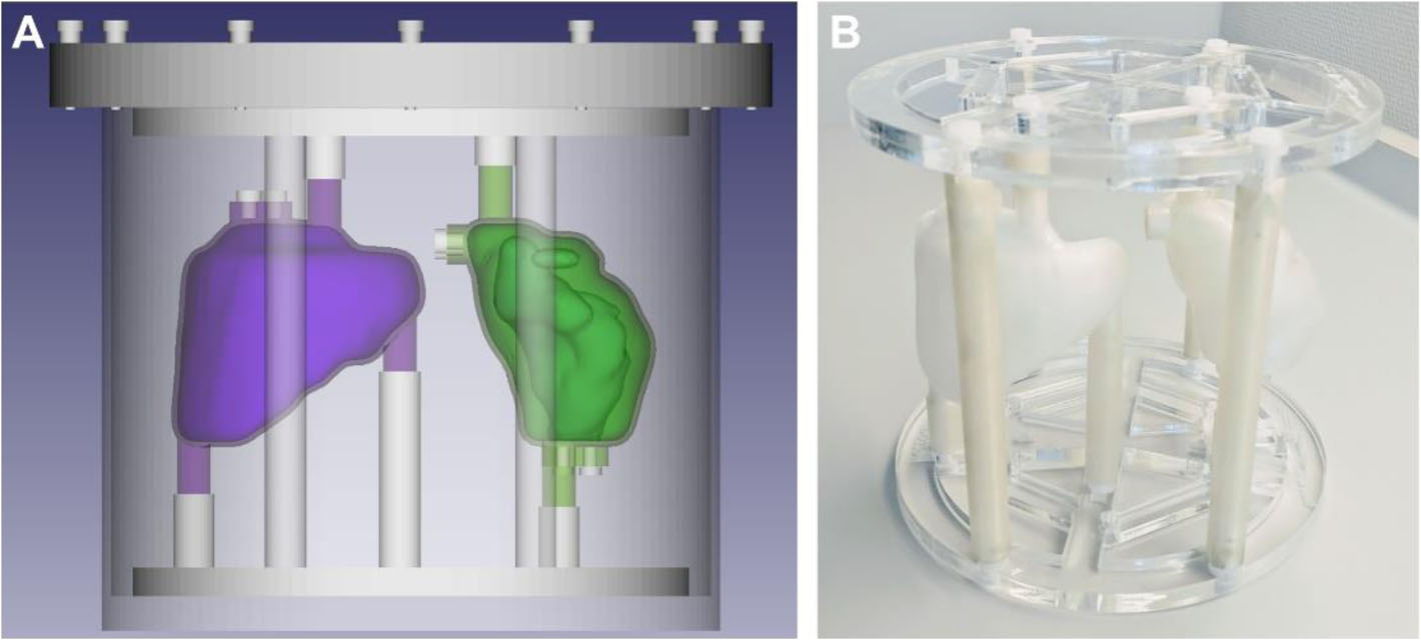
Two-organ ICRP 110 phantom. **A** Computer-aided design (purple: one-compartment spleen, green: two-compartment kidney). **B** 3D printed phantom inserts mounted in the support frame

In the last part of the exercise, the quantitative imaging setup was validated using a 3D printed anthropomorphic phantom (two-organ phantom) modelled based on the adult female kidney and spleen from ICRP 110 [16]. The kidney inserts contained individual compartments corresponding to the cortex and medulla. The inserts were printed with polylactide (PLA) using fused deposition modelling as described in [24–26]. Each centre was equipped with a copy of the phantom designed to be attached inside the previously used Jaszczak phantom using a laser-cut mounting plate and support rods (see Fig. 1).

**Fig. 2:**
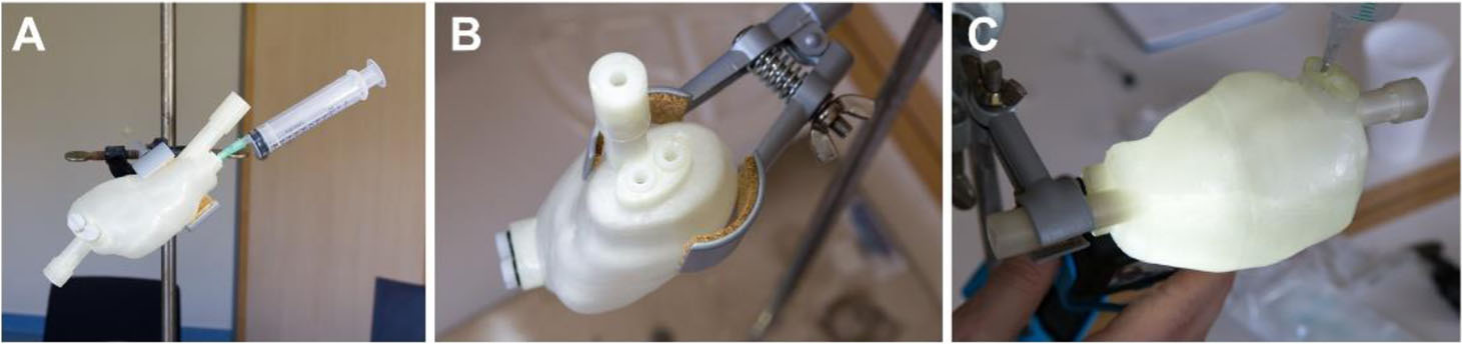
Filling procedure for the kidney insert. **A** Filling of the medulla with the kidney mounted in a clamp stand. **B** Medulla filling holes. **C** Bottom-lit kidney to visualize the filling level

Each organ compartment was filled with a uniform activity of ^177^Lu using two stock solutions to model reduced activity uptake in the renal medulla compared with the cortex [27, 28]. Details of the organ volumes and activity concentrations are provided in Table 3. The filling procedure for the kidney insert is outlined in Fig. 2. SPECT/CT data were acquired with the phantom positioned in the centre of the SPECT field of view using the acquisition parameters described in Table 2.

**Table 3:** Target and dispensed activities and filling volumes for the two-organ phantom inserts with the range of reported values from the comparison exercise

Insert	Target activity (MBq)	Target volume (mL)	Target activity concentration (MBq/mL)	Dispensed activity (MBq)	Dispensed volume (mL)	Dispensed activity concentration (MBq/mL)
Kidney medulla	21	42	0.5	16–22	34–35	0.47–0.59
Kidney cortex	112.5	75	1.5	104–121	74–76	1.44–1.62
Spleen	187.5	125	1.5	171–191	118–125	1.44–1.62

### Image reconstruction

All phantom data acquired during the exercise were reconstructed locally at the sites using common reconstruction parameters with setup-specific choices of scatter correction and resolution recovery (as used for clinical imaging). The SPECT/CT reconstruction parameters are given in Table 4.

**Table 4:** SPECT/CT reconstruction parameters

Reconstruction	Ordered subset expectation maximization (OSEM)
Attenuation correction	CT based
Scatter correction	Triple energy window (TEW) or as per site protocol
Iterations	5, 10, 15, 20, 25, 30, 35, 40, 45, 50
Subsets	2
Post-filter	No
Resolution recovery	As per site protocol

Data were reconstructed by all sites with a range of iterations (see Table 4) to ensure a sufficient total number of counts in the reconstructed image for convergence. For all sites, stable quantification was observed with 2 subsets and 25 iterations, with the total number of reconstructed counts increasing by < 0.7% for higher numbers of iterations. The target activities of ^177^Lu used for phantom measurements in this study were chosen to ensure negligible acquisition dead-time [29]. Decay corrections for the acquired counts (e.g., decay correction of the count number in each projection to the start of the SPECT acquisition) were applied as implemented by the manufacturer for all data to be reconstructed. For all systems, any reconstruction processing options that produce nonlinear responses were not enabled.

### Image quantification

#### Image calibration factor for ^177^Lu

The reconstructed Jaszczak phantom data were used to determine a setup-specific image calibration factor for ^177^Lu:

1 $$ \mathrm{ICF}=\frac{C}{T\bullet {A}_{\mathrm{Calibrator}}} $$

Here, *C* is the counts in the reconstructed image within a cylindrical volume of interest (VOI) corresponding to 130% of the radius and 120% of the height of the phantom, *T* is the acquisition duration (unit: s), and *A*_Calibrator_ (unit: Bq) is the activity dispensed in the phantom. The standard uncertainty (*u*(ICF)) was calculated according to the multiplicative variant of the law of propagation of uncertainty [30]:

2 $$ u\left(\mathrm{ICF}\right)=\mathrm{I} CF\bullet \sqrt{{\left(\frac{u\left(\mathrm{C}\right)\ }{C\ }\right)}^2+{\left(\frac{u(T)}{T}\right)}^2+{\left(\frac{u\left({A}_{\mathrm{Calibrator}}\right)}{\ {A}_{\mathrm{Calibrator}}}\right)}^2} $$

The standard uncertainty in the counts within any volume can be approximated by Poisson statistics and calculated as the square root of the number of counts [31]. The standard uncertainty on activity measurements was provided by each site according to the methods used. A standard uncertainty of 1 s was assumed for all acquisition durations.

#### Partial volume correction

VOIs corresponding to each of the six sphere inserts were drawn based on a sphere of known sphere diameter positioned using the CT. Recovery coefficients were calculated by dividing the SPECT/CT-based activity in each of the spheres of nominal volume *V*_*i*_ by the activity dispensed to the sphere known from the phantom experiment preparation [10, 32]. The recovery coefficients were fitted to a two-parameter model using a weighted nonlinear regression model

3 $$ R(V)=\frac{1}{1+{\left(\frac{\alpha }{V}\right)}^{\beta }} $$

with the Levenberg-Marquardt algorithm in MATLAB 2020a [33, 34]. The weights of the NEMA recovery coefficients were calculated as the reciprocals of the product of the fractional standard uncertainties of the recovery coefficients and the fractional standard uncertainties of the sphere volumes. The weights were adjusted by the uncertainties in the drawn volume to account for a higher degree of uncertainty in the recovery of small volumes calculated according to the analytical approach of the EANM guidelines [7].

#### Validation of quantitative imaging

VOIs corresponding to the spleen and kidney (total, cortex and medulla) inserts were defined for the two-organ phantom data using the CT images (depending on each individual site’s clinical setup, polygon- and threshold-based VOIs were used). The partial volume corrected SPECT/CT-based activity for each organ VOI were then calculated using Eqs. (1) and (3),

4 $$ {A}_{\mathrm{SPECT}}\left({V}_{\mathrm{Organ}}\right)=\frac{C}{T\bullet \mathrm{ICF}\bullet R\left({V}_{\mathrm{Organ}}\right)} $$

where *C* is the total counts in the reconstructed image within the organ VOI of volume *V*_Organ_. The recovery coefficients *R*(*V*_Organ_) were calculated according to Eq. (3). The standard uncertainty in the ratio between the organ activity calculated from SPECT/CT and as measured in the radionuclide calibrator was calculated as follows:

5 $$ u\left(\frac{A_{\mathrm{SPECT}}}{A_{\mathrm{Calibrator}}}\right)=\frac{A_{\mathrm{SPECT}}}{A_{\mathrm{Calibrator}}}\bullet \sqrt{{\left(\frac{u(C)}{C}\right)}^2+{\left(\frac{u(T)}{T}\right)}^2+{\left(\frac{uICF}{ICF}\right)}^2+{\left(\frac{u(R)}{R}\right)}^2+{\left(\frac{u\left({A}_{\mathrm{Calibrator}}\right)}{A_{\mathrm{Calibrator}}}\right)}^2} $$

Here, the uncertainty in the recovery at a given organ volume *V*_Organ_ was calculated following the law of propagation of uncertainties:

6 $$ u(R)=\sqrt{{\left(\frac{\partial R}{\mathrm{\partial \upalpha }}\right)}^2u{\left(\upalpha \right)}^2+{\left(\frac{\partial R}{\partial \beta}\right)}^2u{\left(\upbeta \right)}^2+{\left(\frac{\partial R}{\partial V}\right)}^2u{(V)}^2} $$

The uncertainties in the volumes were determined following EANM guidelines under the assumption of spherical organs and the definition of target VOIs on SPECT imaging to reflect a more realistic clinical situation [7].

## Results

**Fig. 3:**
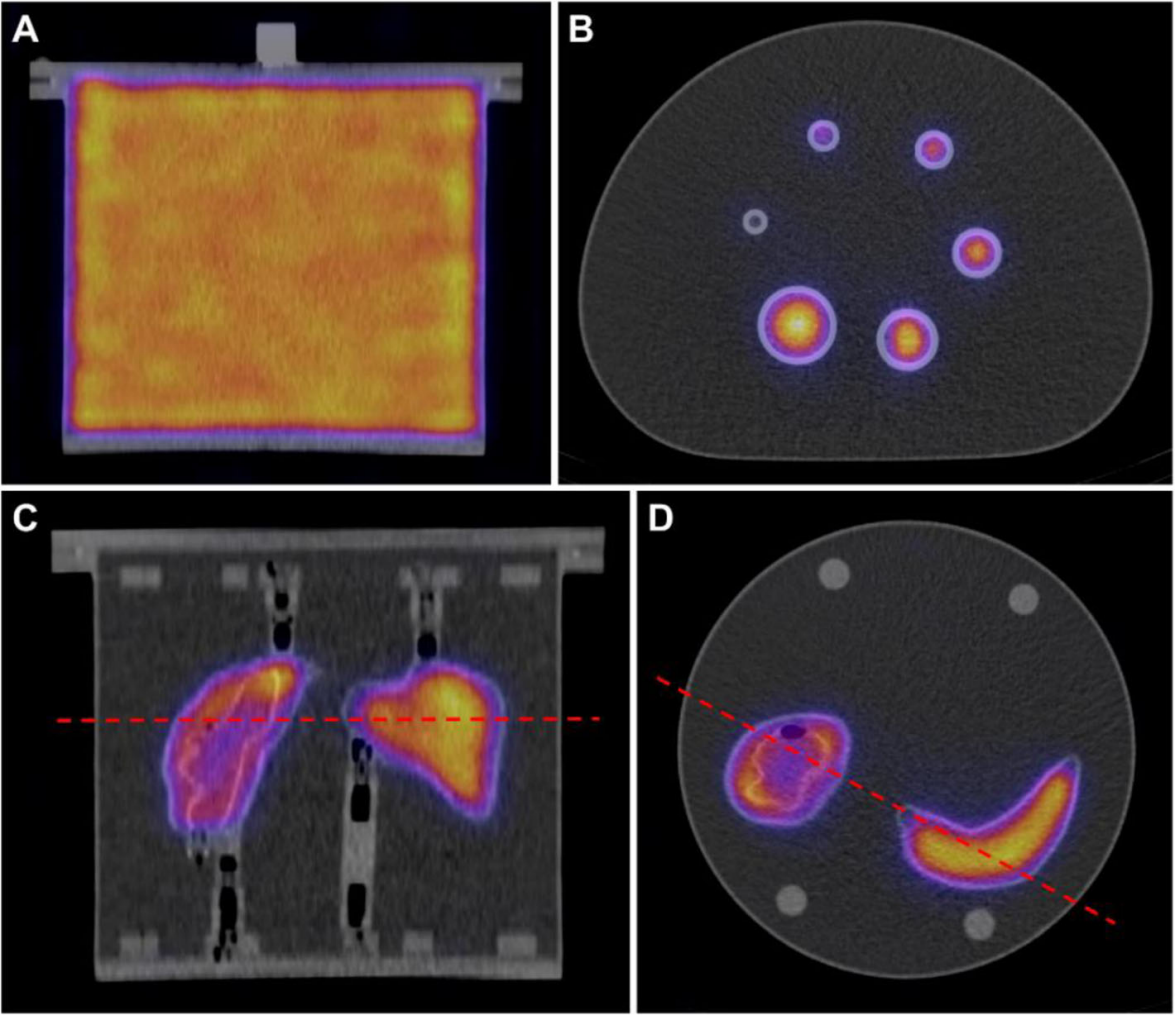
Example SPECT/CT reconstructions for a Siemens Intevo Bold system. **A** Coronal view of Jaczszak phantom. **B** Axial view of NEMA phantom. **C** Coronal view of Two-Organ (kidney and spleen) phantom. **D** Axial view of Two-Organ phantom. The red lines indicate the position of the axial cut for the Two-Organ phantom

A representative example of the reconstructed phantom data is shown in Fig. 3. Each participating centre acquired corresponding datasets for identical phantoms. The centres reported results for phantom activities and counts in VOIs as described in the previous section.

**Fig. 4:**
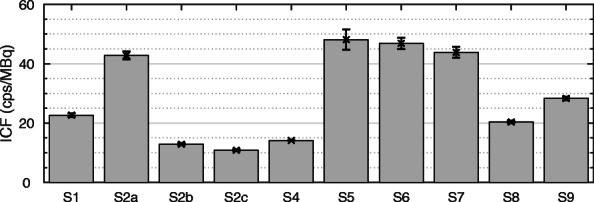
Setup-specific image calibration factors (ICF). Details about the individual setups can be found in Table 1

**Fig. 5:**
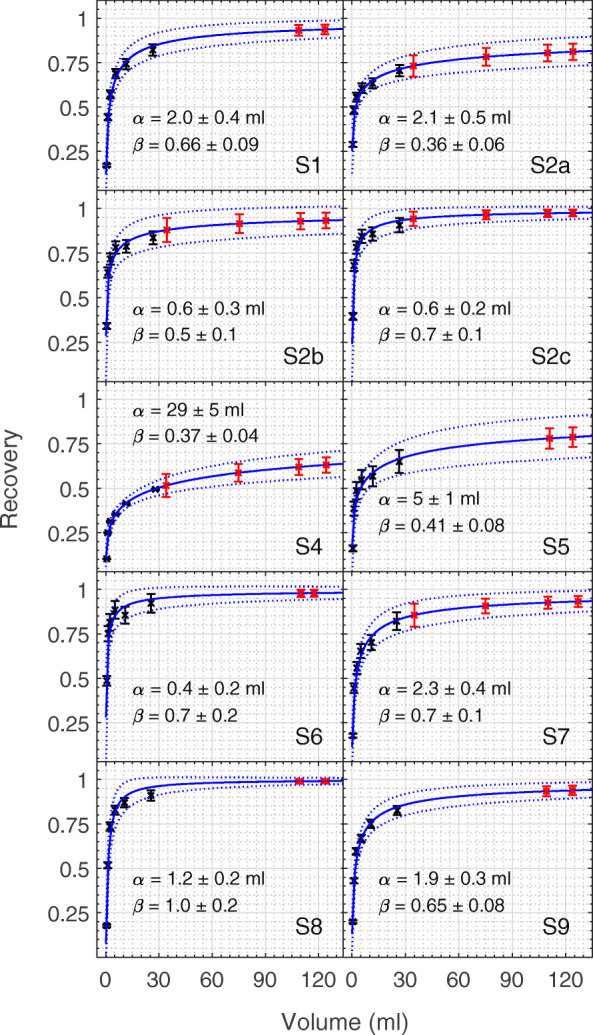
Recovery curves and 95% confidence intervals fitted (to Eq. (3)) using a nonlinear weighted model for the calculated NEMA spheres recovery coefficients (black). The fitted recovery coefficients calculated for the validation data are shown in red

A centralized analysis of ICF, activity recovery and partial volume correction was performed for all results. A total of 10 datasets were included in the comparison. The values of ICF for the datasets are shown in Fig. 4. Results of the partial volume analysis, with the resulting partial volume correction (Eq. (3)) are shown in Fig. 5 for each dataset with the associated 95% confidence intervals. Data points corresponding to the six spheres used for determining the parameters of the recovery curve (black points) and the fitted recovery coefficients for the Two-Organ validation phantom inserts (red points) are shown.

**Fig. 6:**
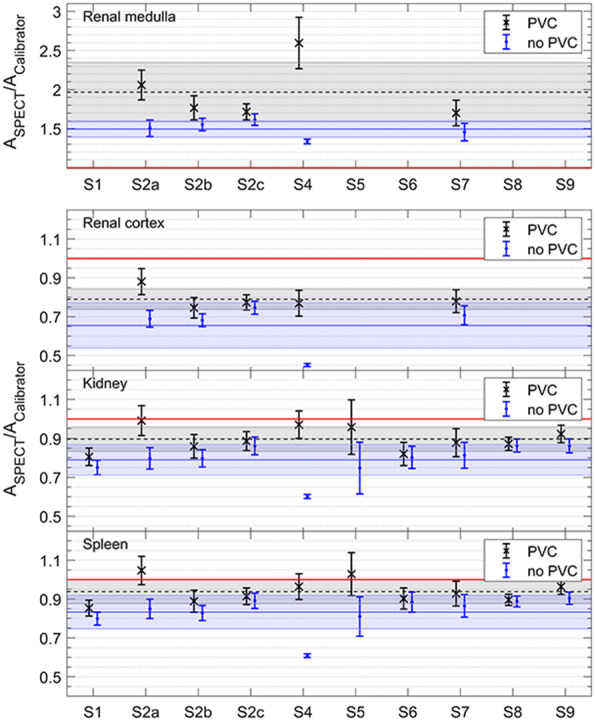
Activity ratios with (black cross) and without (blue circle) PVC applied for renal medulla, renal cortex, kidney and spleen inserts (top to bottom). For each case, the horizontal line shows the average ratio across all setups with the shaded area showing the region within one standard deviation, whilst the red line represents 100% accuracy. Note the different vertical scale for the renal medulla

The ratio of activity determined from SPECT/CT imaging (*A*_SPECT_) to the measured activity in the phantom (*A*_Calibrator_) is shown in Fig. 6 for the total kidney, renal medulla, renal cortex, and spleen inserts. For each setup, data are shown for activity recovery with partial volume correction (PVC) applied (black crosses) and without (blue circles)—see Eq. (3). For each case, the mean ratio is shown as a horizontal line with the shaded area representing one standard deviation. The combined standard uncertainty and contributions from each component are given in Tables 5, 6, 7 and 8 for the renal medulla, renal cortex, total kidney and spleen, respectively.

**Table 5:** Uncertainty budget for renal medulla activity ratios (A_SPECT_: SPECT-based activity, A_Calibrator_: radionuclide calibrator-based activity, R(α) / R(β) / R(V): uncertainty components introduced by the recovery originating from α, β and V, respectively)

Setup	A_SPECT_/ A_Calibrator_	Uncertainty components (%)							Combined standard uncertainty (%)
		Time	Counts	*R*(α)	*R*(*β*)	*R*(*V*)	A_Calibrator_	ICF	
S2a	2.06	0.03	0.05	2.46	4.32	6.54	2.83	3.13	9.23
S2b	1.77	0.03	0.09	2.99	5.65	4.09	2.83	3.13	8.69
S2c	1.72	0.03	0.10	1.31	2.98	2.68	2.83	3.13	5.97
S4	2.60	0.03	0.08	2.95	0.32	12.25	0.52	0.52	12.63
S7	1.70	0.03	0.05	1.78	3.76	6.40	4.25	4.24	9.71

**Table 6:** Uncertainty budget for renal cortex activity ratios (A_SPECT_: SPECT-based activity, A_Calibrator_: radionuclide calibrator-based activity, R(α) / R(β) / R(V): uncertainty components introduced by the recovery originating from α, β and V, respectively)

Setup	A_SPECT_/ A_Calibrator_	Uncertainty components (%)							Combined standard uncertainty (%)
		Time	Counts	*R*(*α*)	*R*(*β*)	*R*(*V*)	A_Calibrator_	ICF	
S2a	0.88	0.03	0.03	1.99	4.47	4.08	2.83	3.13	7.64
S2b	0.75	0.03	0.05	2.11	4.77	2.23	2.83	3.13	7.07
S2c	0.77	0.03	0.05	0.79	2.15	1.25	2.83	3.13	4.96
S4	0.77	0.03	0.06	2.51	1.62	8.03	0.52	0.52	8.60
S7	0.78	0.03	0.03	1.15	3.10	3.20	4.24	4.24	7.56

**Table 7:** Uncertainty budget for kidney activity ratios (A_SPECT_: SPECT-based activity, A_Calibrator_: radionuclide calibrator-based activity, R(α) / R(β) / R(V): uncertainty components introduced by the recovery originating from α, β and V, respectively)

Setup	A_SPECT_/ A_Calibrator_	Uncertainty components (%)							Combined uncertainty (%)
		Time	Counts	*R*(*α*)	*R*(*β*)	*R*(*V*)	A_Calibrator_	ICF	
S1	0.81	0.03	0.04	0.81	2.33	2.10	3.68	2.74	5.61
S2a	0.99	0.03	0.02	1.79	4.44	3.23	4.00	3.13	7.69
S2b	0.86	0.03	0.05	1.78	4.34	1.65	4.00	3.13	7.11
S2c	0.89	0.03	0.05	0.62	1.81	0.86	4.00	3.13	5.50
S4	0.97	0.03	0.05	2.31	2.08	6.52	0.70	0.52	7.28
S5	0.96	0.03	0.02	2.18	5.56	4.12	10.48	7.07	14.58
S6	0.82	0.03	0.02	0.65	1.90	0.67	5.66	4.00	7.25
S7	0.88	0.03	0.02	0.91	2.73	2.24	6.01	4.24	8.21
S8	0.87	0.08	0.06	0.21	0.83	0.48	3.11	2.20	3.94
S9	0.92	0.08	0.05	0.72	2.11	2.00	3.11	2.20	4.85

**Table 8:** Uncertainty budget for spleen activity ratios (A_SPECT_: SPECT-based activity, A_Calibrator_: radionuclide calibrator-based activity, R(α) / R(β) / R(V): uncertainty components introduced by the recovery originating from α, β and V, respectively)

Setup	A_SPECT_/ A_Calibrator_	Uncertainty components (%)							Combined uncertainty (%)
		Time	Counts	R(α)	R(β)	R(V)	A_Calibrator_	ICF	
S1	0.85	0.03	0.03	0.74	2.22	1.86	2.60	2.74	4.82
S2a	1.05	0.03	0.02	1.73	4.42	3.00	2.83	3.13	7.02
S2b	0.89	0.03	0.04	1.68	4.19	1.50	2.83	3.13	6.36
S2c	0.91	0.03	0.04	0.57	1.71	0.77	2.83	3.13	4.65
S4	0.96	0.03	0.04	2.24	2.22	6.05	0.52	0.52	6.86
S5	1.03	0.03	0.02	2.11	5.56	3.84	4.00	7.07	10.77
S6	0.90	0.03	0.02	0.62	1.84	0.63	4.00	4.00	6.01
S7	0.93	0.03	0.02	0.84	2.60	1.96	4.24	4.24	6.87
S8	0.90	0.08	0.05	0.18	0.76	0.41	2.20	2.20	3.23
S9	0.96	0.08	0.04	0.66	2.02	1.78	2.20	2.20	4.17

## Discussion

### Quantitative imaging comparison

The ICF values reported in this work (Fig. 4) are comparable when the same SPECT camera and reconstruction software are used (Siemens - S1 and S8, GE - S2, S5, S6, and S7). The influence of reconstruction software on the ICF is clearly demonstrated by the results for S2a, S2b and S2c where the same SPECT projections have been reconstructed with different software resulting in a large variation in ICF. It should, however, be noted that this apparent reduction in sensitivity is a normalization effect in the reconstruction rather than a change in intrinsic sensitivity. In contrast, the increased ICF value for S9 (compared with the otherwise identical systems S1 and S8) reflects the additional sensitivity from the thicker crystal built into this system. Whilst these variations suggest that a setup-specific calibration is required for accurate QI, the values presented in Fig. 4 can be a useful guide when benchmarking QI calibration of specific system and reconstruction software combinations (see Table 1).

The activity ratio results (Fig. 6) demonstrate significant harmonization of quantitative imaging performance between the sites. For datasets without PVC (blue data points in Fig. 6), all setups are within one standard deviation of the mean values for all inserts, with the exception of S4 which, in contrast to the other datasets, had no resolution recovery applied. When PVC is applied, all setups (including S4) fall within one standard deviation of the mean values.

However, all setups underestimated activity recovery from SPECT/CT imaging with PVC applied for the kidney (mean value 0.90 ± 0.06), spleen (mean value 0.94 ± 0.06) and renal cortex (mean value 0.79 ± 0.05). Overestimated activity recovery was reported by all sites for the renal medulla (mean value 1.97 ± 0.34). There were 3 out of 10 setups with activity recovery within one standard deviation of 100% for the kidney, and 4 setups for the larger spleen. Additional 2 setups (kidney) and 4 setups (spleen) were within two standard deviations. The uncertainty in the final activity quantification (a crucial input to subsequent absorbed dose calculations) was consistent across all datasets with a mean value of 7.2 ± 2.5% for all inserts (see Tables 5, 6, 7 and 8 for details).

Whilst the establishment of a common protocol has clearly demonstrated harmonization across the datasets, the accuracy of the resulting activity recovery was found to be limited by the choice of PVC. This is not unexpected with previous studies highlighting both the importance of accurate PVC for QI [10] and the difficulty of implementing more advanced algorithms in a clinical context [35]. Specifically, the choice of relatively small spheres for the partial volume determination (volumes < 30 mL in comparison to volumes of > 100 mL used for validation) limits the accuracy of the PVC. This decision was made with the aim of focusing on the most commonly clinically available phantoms. However, larger size inserts may be advantageous to improve the accuracy of the PVC methodology for larger volumes. In this current study, it is notable that for all setups, the application of partial volume correction improved activity recovery for inserts where no activity was present outside most of the insert. The mean increase in activity recovery was 0.11 (kidney), 0.13 (renal cortex) and 0.11 (spleen). In contrast, activity recovery was significantly overestimated with PVC when activity was present outside the insert, with an average increase of 0.47 for the medulla. This highlights the limitations of any volume-based PVC method where there is significant spill-in from outside a VOI as for the kidney medulla, which is enclosed by the kidney cortex with a 1.8-fold larger volume in combination with a 3-fold higher activity concentration than the medulla. In these cases, no volume-based correction method will manage to yield an exact correction.

The data for kidney and spleen obtained without resolution recovery in case of S4 allows a direct comparison with theoretical and experimental activity recovery values reported in previous work [24, 25, 36]. The values for the kidney (0.60 ± 0.01) and spleen inserts (0.61 ± 0.01) agree (within measurement uncertainties) with values reported by [25] for kidney and spleen inserts corresponding to mathematical models from [37] for measurements with a GE system. The values for the kidney inserts also agree with the values reported in [36] for patient-specific inserts based on CT imaging. A similar trend in activity recovery for ^177^Lu in kidney phantom inserts corresponding to models from [38] is reported in [24] for measurements with a Siemens system. Repetition of this comparison exercise for data reconstructed without resolution recovery would allow the generality of these findings to be further investigated.

In general, whilst the application of resolution recovery increases activity recovery for the organ-sized volumes in this study, all data still require additional PVC (see Fig. 6). For the medulla, however, spill-in from the cortex leads to an overestimation of the activity concentration which is then further increased by the partial volume correction. In fact, it is notable that the application of PVC without resolution recovery (S4) is one out of only 3 setups with activity recovery within one standard deviation of 100%, in contrast to the majority of setups where PVC was applied to data with resolution recovery already applied. However, it should be noted that for data that include resolution recovery, the uncertainty contribution from the PVC (R(α), R(β), R(V) in Tables 6, 7 and 8) is substantially smaller than for data without resolution recovery. This reflects the better convergence in terms of activity recovery for smaller volumes, as seen in Fig. 5, resulting in an order-of-magnitude reduction in the fit parameter *α* when compared with S4, with a corresponding increase in the sensitivity of the volume component of the uncertainty, *R*(*V*), for S4. It should also be noted that S4 reported an order-of-magnitude lower uncertainty for the activity measurements compared with all other setups (average 0.6% compared to 4.0% for other setups), which balanced the increased PVC uncertainty in the final combined uncertainty. These results clearly demonstrate the complex interconnection between corrections used for quantitative imaging and the benefits of a comprehensive uncertainty analysis for understanding and prioritizing these corrections.

### Protocol for commissioning SPECT/CT QI

The quantitative imaging comparison performed in this study has demonstrated that harmonization of quantitative SPECT/CT imaging across multiple international sites is feasible when following a common protocol. Ensuring traceability of activity measurements to an agreed primary standard is an essential underpinning step to obtain accurate quantitative radionuclide imaging. The protocol set out for commissioning QI in this study is self-contained, requiring no previous setup for ^177^Lu QI at a site, and provides clinically relevant validation of QI for a specific scanner.

This study has demonstrated that the absolute accuracy of SPECT/CT QI is highly dependent on the PVC methodology. Whilst this dependence may present challenges in validating QI at a single site following this protocol, the robustness of the results presented in this study provides a valuable benchmark for sites commissioning SPECT/CT QI for ^177^Lu. As such, the adoption of a common (although unoptimized) PVC methodology for validation of QI has clear benefits. After such validation, further optimization of PVC and other imaging corrections, applied identically to both calibration and clinical imaging and based on local clinical requirements and SPECT/CT equipment, may be beneficial.

Adopting common clinically realistic test objects for validation such as the two-organ phantom used in this study, gives increased confidence in QI commissioning and cross-site harmonization. Such test objects can also be powerful in understanding the limitations of QI methodologies (as demonstrated by the significant overestimation of activity in the kidney medulla observed in this study) and their potential optimization. The reported designs for the two-organ phantom (in the STL file format) are offered as a freely available option for validation [17].

The methods for SPECT QI outlined in this study can provide the basis of a calibration and commissioning protocol, supported by the presented inter-comparison results and phantom designs. A next step will be to define a protocol for commissioning SPECT/CT QI, which utilizes the baseline results from this publication to allow validation of single site SPECT/CT QI for ^177^Lu. The adoption of such a protocol is an essential step in supporting larger-scale clinical trials involving SPECT QI.

## Conclusion

This comparison exercise shows that reliable quantitative SPECT/CT is feasible when following the very specific recommendations of the dedicated calibration protocol developed within the MRTDosimetry project. It is of high value as it was conducted in an international setting, bringing together the expertise of clinical sites and metrology institutes to ensure traceability and assess uncertainties in the activity determination—two aspects that are rarely considered in comparison exercises in the field of nuclear medicine. For an anthropomorphic two-organ phantom (ICRP kidney and spleen), the count loss due to spill-out was successfully compensated by using a standardized partial volume correction based on sphere recovery coefficients. In conclusion, this work shows that, given a detailed and standardized protocol for the measurements to be performed, quantitative SPECT/CT systems can be commissioned to deliver comparable activity values which is important for larger-scale clinical trials.

## Data Availability

To allow clinical centres to implement the techniques presented in this work and benchmark QI against these results, the standard operating procedure for the comparison exercise, the designs used for the phantom fabrication (in the STL file format), and the designs used for the phantom fabrication (in the STL file format) have been made available [17]. The datasets generated and analyzed in the course of the comparison exercise are available from the authors on reasonable request.
